# Women’s experiences of surviving severe obstetric complications: a qualitative inquiry in southern Ghana

**DOI:** 10.1186/s12884-022-04538-w

**Published:** 2022-03-16

**Authors:** Ruby Elikem Afi Amegavluie, Mary Ani-Amponsah, Florence Naab

**Affiliations:** 1 37 Military Hospital, Intensive Care Unit, Accra, Ghana; 2 Maternal and Child Health Department, School of Nursing and Midwifery/ College of Health Sciences. University of Ghana, Legon, Ghana

**Keywords:** Severe obstetric complications, Survive, Women, Experiences, Ghana

## Abstract

Women who survive severe obstetric complications (SOC) have health and well-being issues even up to 1 year postpartum and have challenges in their quality-of-life (QoL). However, little is known about their predicaments. This study aimed to describe the impact of severe obstetric complications on women’s QoL and well-being after surviving severe obstetric complications. Using the WHO standards for near-miss, twelve (12) women who survived severe obstetric complications were recruited between January and March 2019. The study adopted a qualitative approach with an exploratory descriptive design to explore the experiences of women who survived SOC in Southern Ghana. The participants were purposively sampled and were interviewed face to face in their homes and healthcare facility after discharge from the Hospital. Recorded interviews were transcribed and analyzed. Two (2) major themes and nine (9) sub-themes emerged. The findings revealed that women who suffered SOC are unable to perform functional activities, have financial constraints, residual hypertension, signs and symptoms of anaemia, pain, and mostly have difficulty in sleeping due to fear of death when they fall asleep. Anxiety, sadness, and emotional trauma was a common phenomenon. The study findings offer insights and directions on measures to improve the care and QoL of women who have survived severe obstetric complications in Ghana.

**Supplementary Information:**

The online version contains supplementary material available at 10.1186/s12884-022-04538-w.

## Background

Pregnancy, usually seen as a normal process, is now frequently experienced as a period of real or potential risk to both the expectant mother and the unborn child [1]. Although majority of women believe that pregnancy is a joyful and blissful period in their lives, the hassles and changes that accompany it can produce undesirable experiences for some expectant mothers [2]. Severe obstetric complications include Pre-Eclampsia/Eclampsia, Haemorrhages, syndrome of haemolysis, elevated liver enzymes and low platelet count (HELLP syndrome). Also, thrombosis, threatened abortions, uterine rupture, gestational diabetes, placental praevia/infarction, cephalo-pelvic disproportions, congenital malformations, cord prolapse, prolonged labour, among others are some of the complications of SOC [3]. An obstetric complication is said to be any new pathological changes that are experienced by an obstetric patient with a worsening severity and the manifestations of higher number of signs and symptoms which are related to her state of pregnancy [4]. Recently, the World Health Organization’s [5] defines dangerous or life-threatening, severe and acute obstetric complications within the concept of near-miss, as “a woman who almost died but survived a complication that arose during pregnancy, childbirth or within 42 days of termination of pregnancy [6]. Hence, severe obstetric complications (SOC) and maternal near-miss event will be used interchangeably.

The physical well-being of these women in relation to disease, treatments they have received and its impact on other general bodily concerns like pain, discomfort, inability to sleep, and fatigue can affect their activities of daily living, and hence their quality of life. Prada, Bankole [7] posited that the overall physical health of women who survive SOC tends to be affected. Also, the most common health consequences of women with abortion related near-miss experience were severe abdominal pains and bleeding. A study by Lindqvist, Persson [8] found that severe injury to anal sphincter muscle during childbirth may cause physical and psychological complications and suffering which include pain, urinary and faecal incontinence. LaCross, Groff [9] discovered that women with third-or-fourth-degree perineal laceration leading to faecal incontinence have poor emotional health and depression postnatally [8]. According to [10], these problems may lead to social isolation and changes in sexual intimacy, feelings of guilt, shame, frustration and poorer quality of life even up to 1 year postpartum.

The psychological well-being of women who experienced SOC is influenced by internal and external factors. Both qualitative and quantitative studies done in high-income as well as low-and-middle-income countries found psychological distress, episodes of depression, emotional stress, ruminative thoughts, self-blame, sadness and anxiety to be some of the consequences of SOC Assarag, Dujardin [11]. Besides these negative experiences which affect their psychological well-being, some women tend to have positive attitudes, especially when they have live babies and have survived the severe complications [12].

In Ghana, women who survive SOC face many challenges, however, very little research has been conducted on their experiences of surviving SOC. Therefore, this study explores the experiences of women who survived severe obstetric complications or had near-miss experiences of SOC but were saved due to prompt medical interventions.

## Methods

### Study setting

The study was conducted at the 37 Military Hospital, Accra. The 37 Military Hospital is a quasi-governmental and specialist hospital in Accra, Ghana. It is the third largest referral and teaching hospital in Ghana after Korle-Bu and Komfo Anokye Teaching Hospitals with about 3000 deliveries annually. Eclampsia and hypertensive disorders of pregnancy are the leading cause of maternal mortality in the facility. The hospital provides 24-h maternity services to clients across the country.

### Inclusion criteria

The criteria for including participants in this study were:

- Women who have survived severe obstetric complication from time of discharge from the health facility up to 1 year postpartum.
- Women who are 18 years and above.
- Having survived any one of the severe obstetric complications using the WHO near-miss inclusion criteria.
- Residing within the Accra Metropolis.

### Sampling technique and sampling size

A purposive sampling technique, which is a non-probability sampling technique in which the samples are selected based on the characteristics of the population and the objective of the study [13] is used. Twelve (12) women who survived severe obstetric complications and had been discharged from the health facility were recruited and enrolled into the study between January and March 2019 after their informed consent was obtained. The researchers selected participants until data were saturated. Participants were informed about the voluntary nature of the study and given information on the right to refuse to take part or opt out of the study at any point in time without being punished or being refused their healthcare.

### Data collection

Ethics approval was obtained from the institutional ethical review board (IRB) of Noguchi Memorial Institute of Medical Research and 37 Military Hospital (NMIR-IRB CPN 026/18–19). All participants were purposively selected based on their near-miss experiences. Most of the data were collected in the participants’ own homes and others at a suitable place within the healthcare facility which was convenient for both the researchers and participants. Face to face in-depth interviews were conducted using an interview guide. In-depth interviews have the advantage of getting detailed information which is full and rich from the subjects [14, 15]. All the interviews were audio recorded on participant’s permission. Each interview session lasted between 45 min and 60 min. All the recorded interviews were transcribed verbatim. Data were collected at the women’s convenience at the healthcare facility, and some of the interviews were held in participants’ own homes. With the use of open-ended questions, the participants were given the freedom to share their experiences freely on the phenomenon under exploration [16]. The use of observational method also made it possible for the researchers to record behaviours like facial expressions and gestures as they occurred [17]. In addition, reflective journal and field notes were also kept as sources of the research data.

### Methodological rigor

Rigor was maintained using the four criteria: credibility, dependability, transferability and confirmability in order to ensure trustworthiness of the study [18].

The study was peer reviewed by all researchers and the professionals in the area of research, this was to ensure credibility of the study. Triangulation was done using multiple methods of data collection by the use of semi-structured interview guide (Additional file 1: Appendix A), observing gestures, facial expressions and recorded in field notes [19].

A pretesting of the research instrument was done in a Hospital in Accra to assess the reliability of the interview guide. An interview of four [4] women who have survived SOC was done in order to ensure dependability. The results of the pretesting informed the researchers to review the research instrument to suit the research questions.

Transferability is the degree to which the results can be generalized or transferred to other settings. It denotes the applicability of research findings to other locations or groups with similar conditions [19, 20]. The researchers described the data collection process, tools, and sampling size in order to ensure transferability.

Confirmability is the researcher’s ability to establish that the data collected truly represents the participants’ responses and not the researcher’s viewpoints/biases [20]. The researchers ensured confirmability by describing to readers and future researchers how findings, conclusions and interpretations were established from data of participants. The researchers ensured this by providing rich quotes from the participants’ data and how each theme emerged from the data gathered.

### Demographic data

All the participants had between two (2) and eight (8) people living with them in their households. They all experienced various forms of SOC within Southern Ghana. Out of the 12 participants, 5 were referrals from other health care facilities within the Southern Ghana to the 37 Military Hospital. All the 12 participants experienced either one (1) or two (2) of the following near-miss conditions; Severe haemorrhage, Eclampsia, uterine rupture leading to interventional hysterectomy and complications of abortion. The details of the sociodemographic characteristics are illustrated in Table 1.

**Table 1:** Demographic Data

Age	Marital status	Educational background	Occupation	Religion	Cause of complication	Lost Baby
29	Married	Masters Degree	Manage an eye clinic	Christian	Haemorrhage/Blood Transfusion	No
35	Married	SSCE	Health Assistant	Christian	Eclampsia	Yes
41	Married	SHS	Trader	Christian	Haemorrhage/Ruptured Uterus/Hysterectomy	Yes
29	Cohabiting	Bachelor Degree	Radiographer	Christian	Haemorrhage/Blood Transfusion	No
35	Married	SHS	Trader	Moslem	PIH/Ruptured Uterus/Hysterectomy	Yes
37	Married	HND	Operations Officer	Christian	Eclampsia	No
28	Married	Tertiary	Community Health Nurse	Christian	PIH/Pulmonary Embolism	No
38	Married	Tertiary	Teacher	Christian	Eclampsia	Yes
33	Married	JSSCE	Trader	Christian	Eclampsia	Yes
39	Married	SSCE	Trader	Christian	Eclampsia/Renal Dialysis	No
22	Boy Friend	SSCE	Student	Christian	Septic Abortion/Laparotomy	Yes
36	Married	SSCE	Sales Personnel	Christian	Pre-Eclampsia/ Gaped C/S wound	No

### Socioeconomic and reproductive health indicators of women interviewed

#### Data analysis

All the participants were given pseudonyms to ensure confidentiality of the data collected. The data analysis was done concurrently with data collection to allow for adjustment of questions on the study instruments. This was done by reviewing the questions for subsequent data collected in view of the response to research questions posed through a meticulous process of data familiarization, coding, theme development and revision. The data were analyzed using thematic analysis framework techniques. The digitally recorded data were transcribed into text and thematically analyzed by the researchers. Themes and sub-themes that emerged were identified by the researchers, discerning similarities and differences between women with SOC and making connections between the themes. It was realized that women who experienced loss of stamina also complained of having weakness and pain. New themes that emerged were added until all the transcripts were analyzed. It was established that, loss of stamina and weakness experienced by these women were due to the SOC they suffered. The interpretation of the findings was done, and conclusions were drawn based on the frame of reference guided by the research questions. The two major themes which emerged were a). Physical wellbeing and b). Psychological Wellbeing.

The details of the (two) major themes and nine (9) sub-themes are described in Table 2.

**Table 2:** Themes and Sub-themes

Themes	Sub-themes
**1. Physical Well-being**	i. Lack of stamina and weakness ii. Residual illness iii. Pain iv. Sleep disruption v. Permanent infertility
**2. Psychological well-being**	i. Feeling sad and worried ii. Loss of confidence for subsequent pregnancies iii. Emotional Trauma iv. Lack of knowledge of condition

#### Findings

From the data collected, 10 out of the 12 women interviewed were married, and 11 women were also working with at least a Junior High Secondary School education certificate. A majority of the women suffered eclampsia as the leading cause of their predicament; this was followed by postpartum hemorrhage.

### Accounts of SOC

#### Physical well-being

The physical well-being of all participants of the study were impacted by the experiences of the severe complications they survived as their physical well-being was compromised in one way or the other.

#### Lack of stamina and weakness

In the participants’ narratives, they revealed that they are unable to engage in any hard work as compared to previously. Some of them revealed that they have lost their strength and stamina for their usual functional activities of household chores, and that they need to be assisted before they can perform such activities. For example, some women could not even carry their own babies due to pain and feeling of weakness as they have not yet fully recovered from their ordeal. They recounted the following:

For instance, today when I was coming here, I had some goods I have been selling and I wanted to bring some for sale, I did all that I could but couldn’t carry the bag with goods, so I left it at home and came without it. I just left it on the bed and came here. Hmmm! Oh! I don’t have enough strength and energy to do things by myself. I felt so dizzy and then just sat down quietly. Oh! It is not easy at all, at first, I could do everything by myself but now, not at all. **(35 years old, near-miss, hysterectomy, still birth).**

Eeerrr!, currently I can’t do any hard work, yes, because things like lifting of heavy things, washing and all those hard household chores is like somebody has to be doing it for me now. And this thing affected me to the extent that I couldn’t even carry my own daughter. **(29-year-old, near-miss, live birth).**

Since after my discharge I have realized that when I talk too much, I become tired and, I cannot stand heat at all and when I go to a place that there is heat, I become very uncomfortable and uneasy. Also, my heart beats very fast when I walk a short distance and now, I cannot do too much of my household chores because I easily get tired when I try to do that. **(36-year-old, eclampsia, perinatal loss).**

#### Sleep disruption

The rest and sleep pattern of some of the participants were affected following severe obstetric complications experienced. While some of the women could not sleep due to fear of dying, others could not sleep simply because their babies cry at night which disrupts their sleep.

Since I was discharged from the hospital (ICU), I had difficulty in sleeping because almost 1 week in the ward from the ICU, I was so much afraid that if I close my eyes, I wouldn’t be able to wake up again, I might die in my sleep, so I didn’t sleep. **(28 years old, near-miss, live birth).**

My baby can also worry me at night by crying a lot and during the daytime too she doesn’t sleep so I must see to the baby until she sleeps before I can also sleep, and this has been a major challenge regarding my sleep. **(28 years old, near-miss, live birth).**

**A** 35-year-old who survived Eclampsia narrated her experience as follows:

Since I delivered, I have not been able to have a good night sleep, whenever I tried sleeping, I remember the serious challenges I faced during labour which made me almost lose my life, I think I need a psychologist before those memories comes whenever I want to sleep. **(35-year-old, near-miss, still birth).**

### Residual illness

It was realized that the women had issues with their overall physical health as a result of the near-miss event. The overall physical health of some of the participants had improved as the days went by however, others were compromised due to the severe nature of the complication they survived. Some women who suffered hypertensive disorders of pregnancy which persisted after the pregnancy and others exhibited signs and symptoms of anaemia for example, dizziness, pale conjunctiva, lips, and palms following their SOC experiences. For instance, these participants described their ordeal:

I felt dizzy sometimes and when I went for check-up, they tell me my B/P is too high. I was not a hypertensive patient, I just experienced this high B.P during the pregnancy which I was given medications, so I thought it will subside, but since delivery, whenever I go to the hospital, they tell me my B.P is high. I do not know what to do. **(35 years old, potentially life-threatening condition, hysterectomy, still birth).**

Following the pregnancy, I have been looking pale and feeling weak, sometimes I feel like passing out. I have been visiting a diagnostic center nearby and the information I always get is that your hemoglobin level is low. **(36 years, near-miss, live birth).**

A 33-year-old woman who survived Eclampsia also narrated:

My only problem was the high B/P which made my heart to beat very fast when I walk a lot. So, since I left the hospital, my general health has not been as how it was at first, I felt very weak and tired. **(33 years old, near-miss, still birth).**

### Pain

Some women specifically reported having pain at the incisional sites:

It’s like menstrual pain, it starts with cramps, so painful that when it starts, you cannot do any other thing. When it happens like that, I don’t wait for it to become severe. I take the next dose of my pain medication to prevent the severity of the pain. **(29 years old, near-miss, live birth).**

Eeerrh! Since that time, I still limped. I delivered in July last year is about 6 months now and I still have the pain in my right knee and I still limp. I think it is because of the complications I experienced because this did not occur which my first pregnancy **(35 years old, life-threatening condition, still birth).**

After the complication I experienced, I have been having chest pains, may be because I have been thinking lately a lot about the whole situation and how I lost my baby. **(39-year-old, near-miss, perinatal loss).**

### Permanent infertility

In addition to the pain experienced by the survivors of the near-miss complication, the fertility of most of the participants has also been compromised due to the complication they have suffered. Some of the women who had no children and however, had their uterus removed due to ruptured uterus during labour in order to save their lives.:

Hmmm! I have not been having my menses again. They told me that when they were doing the C/S they found out that my uterus had ruptured and was beyond repairs, so they took it out. The doctor told me that since he has removed my uterus, I would not be able to have my menses again. **(35 years old, near-miss, still birth).**

A 38-year-old woman who survived Eclampsia also recounted how she had lost her fertility and her baby:

The doctor told me after the C/S that I cannot give birth again because I had a lot of complications and due to that they had to remove the baby and my uterus to save my life. Hmm so sad, I have lost two important things that are so dear to my heart. **(38 years old, near-miss, still birth).**

I don’t have a uterus again and I have lost my baby too…... (Sobbing with tears in her eyes) my doctor said she wanted to repair the uterus for me but the way it had ruptured it was difficult and she couldn’t repair it. Also, she told me the way I was bleeding too, if they try to repair it, they will lose me, so they must take it out. **(41 years old, near-miss, still birth).**

### Psychological well-being

The women had their psychological well-being compromised in one way or the other due to the severity of their condition which has created anxiety and discomforts in them. Other women who experienced traumatic delivery and perinatal deaths had a lot of anxiety and concerns about a potential or actual harm to their babies. This experience influenced their way of perceiving the predicaments they experienced. They recounted having levels of anxiety due to the severe obstetric complications they experienced, however, those who lost their babies through the process experienced heightened levels of anxiety, sadness, and worry. Also, some of the women who lost their babies but did not get the opportunity to see the dead baby experienced less anxiety.

### Feeling sad and worried

Some women narrated what made them sad and worried:

I was asking of the baby after the operation, not knowing the baby is dead, so they brought the dead baby for me to look at. I touched her and she didn’t cry, talked to her abut no response so I became sad, but this got worse when I came to the ward and everybody who had delivered were holding their babies and mine wasn’t there, I became very sad and worried. **(41 years old, near-miss, still birth).**

Other women had heightened anxieties and psychological trauma by seeing other colleagues with their live babies and by explaining to the people around them about their predicament of stillbirth.

I was worried when I was in the ward seeing other mothers holding their babies, also when I went home people saw me and congratulated me (‘Wutri nkwah’ in Akan language meaning congratulations) and asked how the baby was doing? These made me feel very uneasy, worried, and sad because at every instance, I must repeatedly tell them my baby did not survive it. **(33 years old, life-threatening condition, still birth).**

Sometimes I can no longer hold my tears when I see some friends and relatives playing with their children, because that was my first pregnancy (**41 years old, near-miss, still birth).**

**A** 39-year-old who survived SOC also has this to say:

My problem is money. We spent all our savings on this pregnancy, and we ended up losing the baby. The money issues make me feel so sad and worried because we don’t have any support from anywhere and it’s like it is just me and my husband who must shoulder all the economic burden, we are the only people who hustle for our living. **(Jumak R10).**

This notwithstanding, sadness was expressed by some of the women in various forms and due to different reasons. Some recounted becoming sad and depressed upon hearing the news of their babies’ death. Others said the huge hospital bills made them depressed whereas others became very depressed because of their illness. Other participants felt very sad and worried when the doctor informed them about the removal of their uterus in order to save their lives. Some of the women expressed their sadness by avoiding people or associating with society. Others were even referred to the clinical psychologist for management. Some women recounted what the presence of other people meant to them:

When I go out and people start to ask me of my baby, I feel too sad and depressed. Some will ask if the baby is in the house, and I just don’t know what to tell them. Hmmmmm! I don’t know what to do. So, I have chosen to wear black clothes, so if they see me wearing black they would not ask me of my baby, and I prefer that. **(41 years old, near-miss, perinatal loss).**

Some of the women had other issues that made them feel depressed:

I was recovering but when I remember my 2 sisters, whom one was getting married and the other one had a baby, and it was like everybody’s life was moving forward I cannot afford but to cry. **(29 years old, near-miss, live birth).**

Nevertheless, most of the participants were not able to control their feelings of depression, anger and distress which made them to be emotionally imbalanced.

### Loss of confidence for subsequent pregnancies

Some of the women experienced emotional trauma because of the events or experiences of SOC that made them felt unsafe and helpless. Due to this, most of the women who had their uterus intact vowed not to get pregnant anymore.

As for me, I have decided that I will not give birth again in any way, because I would not like this complication to happen to me again, it wasn’t a good experience at all. It has delayed and stagnated most of my progress. Besides, I think four (4) children are enough, I even wanted to stop at three (3) children, but it has become four (4) now, so it is okay, hahaha! (**39 years old, life-threatening condition, live birth).**

I used to have so much interest in giving birth, but I have lost it after this terrible experience. I have lost the confidence to give birth; I don’t even have the confidence of giving birth again or even going to the operating theatre for any other procedure. Even I am planning to have a tubal ligation and upon a second thought, I realized that even that one too I must still go back to the operating theatre, and I just don’t want to step foot at the theatre again. **(36 years old, near-miss, live birth).**

### Emotional trauma

Some of the women experienced emotional trauma because of the SOC events that have left them feeling empty and helpless. Two women were referred to the Clinical Psychologist for treatment sessions which took the form of debriefing management sessions.

Anytime I remember what happened I felt uneasy, just like when you called me 3 days ago that you were coming to interview me, I was like eeeiii, everything just came back like a movie. Even this morning when you called me that you were coming, I was afraid of saying it again. So, it gives me discomfort whenever I try to talk about it. **(36-year-old, near-miss, live birth).**

The emotional pains I have experienced during the complication were too much and I wouldn’t like to go through a similar experience again so I will no longer have more babies. I have decided I won’t be pregnant again, in fact ever since I came home whenever I see a woman pregnant, I become worried for the person because of my experience. **(35-year-old, near-miss, perinatal loss).**

### Lack of knowledge of condition

Some of the women demonstrated lack of knowledge of the condition they went through:

Where I attended my Antenatal care (ANC), they didn’t explain my condition to me that I had a high B/P so maybe if they had given me some B/P medicine there, I may not have had this complication, because they never gave me any B/P medication. **(35 years old, eclampsia, live birth).**

A 37-year-old who suffered severe Eclampsia also had this to say:

So, I became fat and still had a positive urine protein, but they kept saying I should drink a lot of water and that was the only thing that I was doing. Then elevate your feet, if I keep on doing that the swelling will reduce and I will be fine, that was what the nurse said. **(37 years, life threatening condition, live birth).**

I did not have any complication during my ANC because none of the health care providers noticed that I had a high B/P, it was here that I was diagnosed of it. All that I realized was that I had grown very big (oedema) but I thought that it’s the pregnancy that has made me become very big; I had put on weight. **(41-year-old, near-miss, live birth).**

## Discussion

It is evident from the findings above that survivors of obstetric near-miss lose their strength, stamina, and experience easy fatigue. Angelini, Pacagnella [21] asserted to a similar phenomenon that the health problems borne by women throughout prenatal period, labour and post-delivery period contribute immensely to the overall burden of poor maternal health. The researchers found that almost all the women who survived SOC had some form of health problems after discharge from the hospital. There is evidence that poor health was associated with SOC. Morgan, Henderson [22] and other studies have also identified poor quality of life after the experience of near-miss event [23, 24]. The participants of the study reported various health-related physical well-being issues such as general weakness, residual hypertension, signs and symptoms of anaemia, incisional site pain and general body pain that affected their QoL. This conforms to several studies [11, 21, 25–27].

The overall physical health of survivors of SOC depends on the severity of the complication suffered. The authors again reported that the magnitude of bodily ailment (serious illness) was greater among near-miss cases than deliveries that were uncomplicated. This was cited by several studies [21, 28–30]. In this study, no participant reported to have fever, however, those who survived hypertensive disorders of pregnancy were left with residual hypertension and sleep difficulties. This is consistent to the assertion of Leonard, Main [26] who posited that near-miss survivors tend to have adverse maternal outcomes, coupled with loss of strength and stamina to go about their usual functional activities.

Owing to their life-threatening complications, participants suffered sleep disruptions, difficulties to initiate sleep, and daily face the dangers of insomnia. This conforms to the findings of Annema, Drent [31], Currie [32, 33] and De Cock, Girardot-Tinant [34] that sleeping disorders were common in patients who have newly experienced life-threatening illnesses. Similar to Hinton, Locock [35], Khan, Blum [36, 37], family’s disappointments about the woman’s pregnancy outcome has caused emotional ill health for these women. The study discovered that, women who suffered perinatal loss due to obstetric complications experienced intense anxiety, sadness and worries as compared with those with live babies. This subsequently led them into emotional trauma as the thoughts and perception of death made it difficult for them to discuss challenges associated with their experiences. In this study, some participants have been traumatised to the extent that they would not want to narrate their stories again as it kept reminding them of their ordeal. This supports Elmir, Schmied’s [38] findings which posited that being close to death, bleeding and fear, and having hysterectomy were devastating to these women, as they kept on having flashbacks and memories of their predicaments. This was equally reported by Andersen, Melvaer [39] and Bastos, Furuta [40]. From this study, it could be inferred that SOC was associated with severe emotional trauma.

The study found that traumatic delivery, death anxiety and their concerns about a potential or actual harm to their babies influenced their way of perceiving their predicaments. This compromised the QoL and the psychological well-being of participants. These were sighted in several studies [11, 41, 42]. The researchers again found that women who suffered perinatal loss and interventional hysterectomies had more depression and sadness than near-miss women whose babies survived. In Soma-Pillay, Makin [30] and Kuismanen, Nieminen’s [43] studies, they found that traumatic birth events provoke severe anxiety and distress in both the survivors and their spouses as well as other family members.

The fear of recurrence of the same complications in subsequent pregnancies deterred some of these women and they were hesitant to have future children. They often felt uncomfortable narrating their experience or visiting the hospital, because they lost the confidence to resume their reproductive activities within a reasonable time frame. This conforms to the statements of [30], Andreucci, Bussadori [27, 44] and Moaddab, Klassen [45] who asserted that women who survived life-threatening complications become hesitant to sexual intimacy.

## Conclusion

Obstetric near-miss women have health and well-being issues even up to 1 year postpartum as far as their quality-of-life (QoL) and well-being are concerned. The study found health-related problems such as lack of stamina, general weakness, residual hypertension, signs and symptoms of anaemia, pain, sleeping difficulties, pain, and permanent infertility to be some of the physical well-being issues that affect the survivors of SOC after being discharged from the hospital. This notwithstanding, some of the psychological well-being challenges that survivors of near-miss experience are anxiety, sadness, emotional trauma, and fear of recurrence. It is therefore imperative that women with obstetric complications should be promptly referred to tertiary healthcare facilities to achieve optimal healthcare outcomes. In addition to that, follow up services should be done for women who survive SOC after they are discharged from the hospital as this forms an important medium through which the healthcare providers can find out about their wellbeing, as well as their QoL. There is also a need to conduct weekly maternal near-miss audit and use data to inform and improve practice in all healthcare facilities.

## Supplementary Information

**Additional file 1.** Appendix A.

## Data Availability

All supporting files and data are in the custody of the corresponding author and will be provided upon request.
