# Supplementary material for: Women’s experiences of surviving severe obstetric complications: a qualitative inquiry in southern Ghana
Source: BMC Pregnancy Childbirth. 2022 Mar 16;22:212. doi: 10.1186/s12884-022-04538-w (PMC8928636; doi:10.1186/s12884-022-04538-w)
Supplement: Supplementary file 1 — Additional file 1. Appendix A. [file 12884_2022_4538_MOESM1_ESM.docx]

**Appendix A.**

**Data Collection Instrument** (Interview Guide) – Appendix A

**WOMEN’S EXPERIENCES OF SURVIVING SEVERE OBSTETRIC COMPLICATIONS: A QUALITATIVE INQUIRYIN SOUTHERN GHANA**

**Research Title**: Women’s Experiences of Surviving Severe Obstetric Complications: A Qualitative Inquiry in Southern Ghana.

**Name of Principal Investigato**r: Ruby Elikem Afi Amegavluie (MPhil)

Pseudonym …………………………………………………………………………………

Date of Interview……………………………

Duration of interview………………………….

**Section A**

**Demographic Data**

Age………………………………………………

Religion……………………………………………

Educational Background…………………………….

Ethnicity…………………………………………….

Marital Status…………………………………………

Socio-Economic status ………………………………………

Employment/Occupation………………………………………

Annual household Income……………………………………….

Social support networks…………………………………………..

Type of obstetric complication ……………………………………………

**Section B**

**Obstetric History**

Could you please tell me about your Antenatal care (ANC) history?

**Physical Well Being and Symptoms**

This relates to concerns resulting from the complications experiences, the treatment being received and their impact on other general bodily concerns for example; pain, fatigue, sleeplessness and how it has affected her quality of life.

1. **Functional Activities**

- How has your condition affected your day-to-day living and the performance of your household chores?
- How are you able to survive your activities of daily living?

1. **Strength/Fatigue**

- Could you please give some highlights on the consequences of your diagnosis from the time of discharge from hospital till now?
- How has the complication affected your general health?

Please how has the complication you survived affected yourphysical well-being and quality of life? (Probe).

1. Please concerning the complication you experienced, how did you understand it?

- What does your survival of this condition mean to you?
- To what do you attribute it to?
- Negligence on the part of the hospital staff at ANC or other units
- Personal fault
- Ineffective medications/ inadequate medication
- Complication which will eventually resolve
- Treatment failure
- External forces like someone wants to harm you or God’s will (divine interventions)

1. **Sleep/Rest**

- How has your present health status, childbirth and experience you have been through affected your sleep and rest pattern?
- How do you manage to organize sleep and rest for yourself in the face of your household chores and your baby?

1. **Overall Physical Health**

- What is your overall physical health like after returning home from the hospital?

1. **Fertility**

- Since you were discharge from the health care setting how your menstrual cycle and flow has been like?

1. **Pain**

- What experiences can you share about pain in your abdomen, headache or general bodily aches and how would you describe it?
- How do you describe the pain you are experiencing? And how long does the pain last?
- What do you use to relieve the pain?
- Do you sometimes isolate yourself for various reasons?

1. Please what does the complication you survived mean to you? (probe)

- Meaning do you give to it

1. **Subsequent Pregnancies and associated fears**

- Do you have any fears of achieving subsequent pregnancies? (probe)
- Do you have post-traumatic disorders? (probe)
- Have you lost confidence for subsequent pregnancies?

1. Do you sometimes feel sad and worried?

Please is there any other thing you would like to add?

THANK YOU!!!
